# Aging and Estrogen Status: A Possible Endothelium-Dependent Vascular Coupling Mechanism in Bone Remodeling

**DOI:** 10.1371/journal.pone.0048564

**Published:** 2012-11-20

**Authors:** Rhonda D. Prisby, James M. Dominguez, Judy Muller-Delp, Matthew R. Allen, Michael D. Delp

**Affiliations:** 1 Department of Kinesiology and Applied Physiology, University of Delaware, Newark, Delaware, United States of America; 2 Department of Applied Physiology and Kinesiology and the Center for Exercise Science, University of Florida, Gainesville, Florida, United States of America; 3 Department of Physiology and Functional Genomics, University of Florida, Gainesville, Florida, United States of America; 4 Department of Anatomy and Cell Biology, Indiana University School of Medicine, Indianapolis, Indiana, United States of America; INSERM U1059/LBTO, Université Jean Monnet, France

## Abstract

Bone loss with aging and menopause may be linked to vascular endothelial dysfunction. The purpose of the study was to determine whether putative modifications in endothelium-dependent vasodilation of the principal nutrient artery (PNA) of the femur are associated with changes in trabecular bone volume (BV/TV) with altered estrogen status in young (6 mon) and old (24 mon) female Fischer-344 rats. Animals were divided into 6 groups: 1) young intact, 2) old intact, 3) young ovariectomized (OVX), 4) old OVX, 5) young OVX plus estrogen replacement (OVX+E2), and 6) old OVX+E2. PNA endothelium-dependent vasodilation was assessed *in vitro* using acetylcholine. Trabecular bone volume of the distal femoral metaphysis was determined by microCT. In young rats, vasodilation was diminished by OVX and restored with estrogen replacement (intact, 82±7; OVX, 61±9; OVX+E2, 90±4%), which corresponded with similar modifications in BV/TV (intact, 28.7±1.6; OVX, 16.3±0.9; OVX+E2, 25.7±1.4%). In old animals, vasodilation was unaffected by OVX but enhanced with estrogen replacement (intact, 55±8; OVX, 59±7; OVX+E2, 92±4%). Likewise, modifications in BV/TV followed the same pattern (intact, 33.1±1.6; OVX, 34.4±3.7; OVX+E2, 42.4±2.1%). Furthermore, in old animals with low endogenous estrogen (i.e., intact and old OVX), vasodilation was correlated with BV/TV (R^2^ = 0.630; P<0.001). These data demonstrate parallel effects of estrogen on vascular endothelial function and BV/TV, and provide for a possible coupling mechanism linking endothelium-dependent vasodilation to bone remodeling.

## Introduction

Postmenopausal osteoporosis is characterized by pronounced bone resorption. A clear link between estrogen deficiency and reduced bone mass is evident by the increased prevalence of osteoporosis in women vs. men [1] and the direct association between circulating estrogen levels and rates of bone loss [2]–[5]. In addition, estrogen replacement serves to protect against skeletal fractures and loss of bone [6] primarily by inhibiting bone resorption [7]. The preponderance of literature examining the effects of estrogen status and bone remodeling has attributed the association to the direct effects of estrogen on bone cell activity. However, there are other possible mechanisms whereby postmenopausal bone loss may be influenced and modulated.

There is increasing evidence in the literature that the osseous vasculature influences bone remodeling. For example, vascular endothelial cells release factors such as nitric oxide (NO) and prostacyclin (PGI_2_) that are known to modulate osteoclast and osteoblast activity [8]–[11]. In addition, the osseous resistance vasculature regulates skeletal perfusion and, consequently, fluid filtration and interstitial fluid flow, which can affect bone cell activity through release of autocrine/paracine factors [12]–[17]. Dysfunction of these vascular mechanisms could therefore contribute to postmenopausal and old age-related osteoporosis. Indeed, attenuated bone and marrow blood flow has been reported in healthy humans (>60 years of age) [18], [19], senescent rats [20]–[22] and rabbits [23] and is associated with osteopenia [24]. Old age-related decrements in skeletal perfusion also correspond with diminished endothelium-dependent vasodilation of the femoral principal nutrient artery (PNA), reduced vascular NO bioavailability, and diminished trabecular bone volume and material properties of the femur in male rats [20]–[22]. Thus, aging and estrogen loss may contribute to enhanced bone turnover in females through impairment of endothelial responsiveness in the skeletal circulation.

The purpose of the present study was to determine whether estrogen status affects endothelium-dependent vasodilation in bone blood vessels, and whether this is associated with changes in trabecular bone volume. Estrogen levels were modified in young and old female rats through ovariectomy and estrogen replacement. We hypothesized that the lower estrogen levels in old intact and young and old ovariectomized (OVX) rats would be associated with lower femoral PNA endothelium-dependent vasodilation, and that estrogen replacement in young and old OVX rats would enhance PNA endothelium-dependent vasodilation through a NO signaling pathway. Furthermore, we hypothesized that PNA endothelial vasodilator function would be directly associated with trabecular bone volume.

## Materials and Methods

### Ethical Approval

The procedures used in this study were approved by the University of Florida and West Virginia University School of Medicine Institutional Animal Care and Use Committees and conform to the *Guide for the Care and Use of Laboratory Animals* published by the National Institutes of Health (NIH Publication No. 85-23, revised 1996).

Young (4 mon old) and old (22 mon old) virgin female Fischer-344 rats were obtained from the vendor (National Institute of Aging colony, Harlan, Indianapolis, IN) and divided into six groups: 1) young intact (*n* = 10), 2) old intact (*n* = 11), 3) young OVX (*n* = 10), 4) old OVX (*n* = 9), 5) young OVX+E2 (*n* = 11), and 6) old OVX+E2 (*n* = 10). After arrival all animals were housed for an additional 8 weeks in a temperature-controlled (23±2°C) room with a 12∶12 h light dark cycle. Rats in the latter four groups underwent ovariectomy surgery and were housed post-operatively for 8 weeks. Thus, at the time of sacrifice young animals were at least 6 months of age and old rats were at least 24 months of age. At 6 months of old, Fischer-344 rats are considered sexually-mature young adults, and at 24 months of age are considered senescent when mortality rates are ≥30% [25]. To control for the levels of exogenous estrogen, all rats were provided with a phytoestrogen-free diet and tap water *ad libitum*. Prior to sacrifice, at least two complete estrous cycles were monitored in all female rats by daily vaginal smears. At the time of sacrifice, 3 ml of blood was collected in chilled tubes containing dipotassium EDTA. Blood samples were centrifuged at 10,000 rpm at 4°C for 5 min, and the plasma was collected and stored at −80°C for later analysis. The ovaries (in intact females), uterus and cervix were dissected and trimmed of connective tissue and fat to obtain uterine mass. Plasma estrogen was determined with ELISA immunoassay (Estradiol EIA kit, Oxford Biomedical; Oxford, MI). Rats were sacrificed at 6 and 24 months of age.

### Surgical Procedures

To examine the influence of circulating estrogen on the vasomotor properties of the femoral PNA, the rats were anesthetized with isoflurane (2%/oxygen balance) and subjected to bi-lateral ovariectomy according to standard procedures as described previously [26], [27]. Briefly, bilateral dorsolateral incisions were made through the top layer of skin. The underlying muscles were bluntly dissected until the ovary and fallopian tube were located. For rats in the OVX groups, the fallopian tube was ligated with absorbable suture and the ovary was removed. Once the surrounding fat pad was replaced within the abdominal cavity, the musculature and outer layer of skin was sutured. Estrogen replacement in the OVX+E2 groups was performed simultaneous to the OVX procedure. Two 0.05 mg 17beta-estradiol 60-day slow release pellets (Innovative Research) were implanted subcutaneously near the scapulae.

### *In Vitro* PNA Studies

Rats were anesthetized (isoflurane 5%/O_2_ balance) and euthanized by removing the heart. To assess endothelium-dependent vasodilation of the femoral PNA, *in vitro* experiments were performed. The femur and surrounding musculature was removed and placed in a dissecting dish containing physiological saline buffer solution (PSS) at 4°C. The PNA was isolated from the femur and surrounding muscle tissue by use of a stereomicroscope as previously described [21], [22]. Briefly, the PNA was cannulated onto glass micropipettes, secured with 11-0 nylon microfilament suture (Alcon), and the vessel chamber placed under an inverted microscope (Olympus IX70) equipped with a video camera (Panasonic BP310), video caliper (307A horizontal video caliper, Colorado Video), and data-acquisition system (PowerLab) for the measurement of intraluminal diameter. The PNAs were pressurized to 44 mmHg (60 cmH_2_O) with two hydrostatic pressure reservoirs. This intravascular pressure was selected based on previously reported intravascular pressures of 43±1.8 mmHg and 46±2.6 mmHg in skeletal muscle resistance arteries of similar size to the PNA in normotensive rats [28]. PNAs were then checked for leaks, and those that were leak free were warmed to 37°C and allowed to develop spontaneous baseline tone (≥1 h). Vessels that leaked or did not develop at least 20% spontaneous tone were discarded.

### Evaluation of Endothelium-Dependent Vasodilator Responses

Endothelium-dependent vasodilation was assessed by the cumulative addition of acetylcholine (ACh; 10^−9^–10^−4^ M) to the bathing medium. Prior to each concentration-response protocol, the PNAs developed spontaneous tone. After completion of the experimental protocols, the maximal diameters of the PNAs were determined subsequent to a 30-minute incubation period in calcium-free PSS buffer.

### Evaluation of Endothelium Signaling Pathways

PNAs were cannulated and allowed to develop spontaneous tone. Vasodilation to ACh was evaluated after a 20-min incubation with one of the following: 1) PSS buffer alone, 2) PSS buffer containing the nitric oxide synthase (NOS) inhibitor N^G^-nitro-l-arginine methyl ester (l-NAME, 10^−5^ M), 3) PSS buffer containing l-NAME (10^−5^ M) plus the cyclooxygenase (COX) inhibitor indomethacin (Indo; 10^−5^ M) [21], [22], [29], [30].

### Evaluation of Endothelium-Independent Vasodilator Responses

Endothelium-independent vasodilation was assessed by the cumulative addition of DEA NONOate (DEA; 10^−9^–10^−4^ M) to the bathing medium. Prior to each concentration-response protocol, the PNAs developed spontaneous tone. After completion of the experimental protocols, the maximal diameters of the PNAs were determined subsequent to a 30-minute incubation period in calcium-free PSS buffer.

### Determination of Trabecular Bone Volume

Trabecular bone volume of the distal femur metaphysis was assessed using microcomputed tomography (microCT; Skyscan 1172) as previously described [21]. Femora were wrapped in parafilm to prevent drying during the scanning. Scans were obtained using an x-ray source, set at 60 kV and 167 µA over an angular range of 180 degrees (rotational steps of 0.40 degrees) with a 12-µm pixel size. Projection images were reconstructed using standard Skyscan software. The trabecular bone compartment was segmented from the cortical shell for 50 slices in a region ∼0.5-mm proximal to the most proximal portion of the growth plate for each animal. Images were binarized (threshold of 100–255) and trabecular bone volume/tissue volume (BV/TV) was assessed.

### Solutions and drugs

Stock solutions were prepared in distilled water; whereby ACh was frozen at −20°C and serial dilutions of the ACh stock solution were prepared daily. Serial dilutions of DEA NONOate were prepared fresh as needed. Serial dilutions of the stock solutions were prepared daily. All drugs were purchased from Sigma Chemical (St. Louis, MO).

### Statistical Analysis

Vasodilator responses were expressed as a percentage of maximal relaxation according to the following formula:
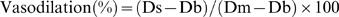
where Dm is the maximal inner diameter recorded at 60 cmH_2_O in calcium-free PSS, Ds is the steady-state inner diameter recorded after each addition of the vasodilator substance, and Db is the initial baseline inner diameter recorded immediately prior to the first addition of ACh. One-way ANOVAs were used to determine the significance of differences in body mass, uterine mass, uterine∶body mass ratio, plasma estrogen concentration, and spontaneous tone and maximal diameter of the PNAs. Repeated measures ANOVAs with pairwise comparisons were used to determine the significance of differences among age and within factors for vasodilator responses. To examine the relation between peak endothelium-dependent vasodilation and trabecular bone volume according to age and estrogen status, linear regression analyses were measured in young and old rats. In addition, regression analyses were performed examining the relations between trabecular bone volume and plasma estrogen, uterine mass, and peak vasodilation in old and old OVX rats. All data are presented as mean ± S.E. Significance was defined as *P≤0.05*.

## Results

### Animal and PNA Characteristics

Body mass (Table 1) was higher in old vs. young intact animals. Body mass was greater in young OVX vs. young intact, and OVX+E2 decreased body mass in both young and old animals compared to age-matched OVX groups. In addition, body mass was lower in old OVX+E2 than in old intact animals.

**Table 1:** Tissue and principal nutrient artery (PNA) characteristics in young and old intact, OVX, and OVX+E2 rats.

	Intact Young	Intact Old	OVX Young	OVX Old	OVX+E2 Young	OVX+E2 Old
*n*	10	11	10	9	11	10
Body mass (g)	211±3	316±5^a^	254±6^b^	324±20^a^	224±5^c^	266±9^a^ ^b^ ^c^
Uterine mass (mg)	644±53	508±24^a^	144±33^b^	386±26^a^ ^b^	339±40^b^ ^c^	728±77^a^ ^b^ ^c^
Uterine∶body mass ratio (mg/g)	3.0±0.3	1.6±0.1^a^	0.5±0.1^b^	1.2±0.1^a^	1.5±0.2^b^ ^c^	2.8±0.3^a^ ^b^ ^c^
PNA maximum diamter (µm)	154±7	167±11	158±13	155±11	183±9^b^ ^c^	187±3^b^ ^c^
PNA spontaneous tone (%)	47±6	39±4	35±4	37±5	33±4	34±3

a Indicates significant age-related difference vs. young of same condition.

b Indicates significant difference vs. age-matched intact.

c Indicates significant OVX-E2 difference vs. age-matched OVX (P≤0.05).

“*n*” indicates number of animals. Values are mean ± SE.

Plasma estrogen concentration for some of the animals in the old intact (5 out of 11), young OVX (1 out of 10) and old OVX (5 out of 9) groups was below the detectable range (5 pg/ml) for the ELISA immunoassay technique used to measure estrogen levels. Therefore, the estrogen concentrations reported for these three groups are an overestimate of the actual levels because estrogen values for these animals could not be included in the analysis. For this reason, we provide median and range values, as well as report uterine mass as a surrogate marker for estrogen concentration. Plasma estrogen concentration in young intact animals (median 62 pg/ml; range 26–103 pg/ml) was greater than that in old intact rats (median 25 pg/ml; range 18–36 pg/ml). OVX decreased plasma estrogen in young rats (median 22 pg/ml; range 6–32 pg/ml), but did not alter estrogen levels in old rats (median 34 pg/ml; range 18–49 pg/ml). In young animals OVX+E2 increased plasma estrogen (median 40 pg/ml; range 35–54 pg/ml) above that in the young OVX group and to a level similar of that in the young intact group. In the old rats OVX+E2 increased plasma estrogen concentration (median 51 pg/ml; range 31–78 pg/ml) to levels above that in both the old intact and OVX groups. Uterine mass (Table 1) was lower in old intact vs. young intact rats. OVX decreased uterine mass in both age groups relative to age-matched animals. OVX+E2 elevated uterine mass in both age groups relative to age-matched OVX rats. Uterine mass was also greater in old OVX+E2 than in old intact animals, whereas uterine mass was lower in young OVX+E2 relative to young intact. Age and condition effects on uterine∶body mass ratio were similar to those of uterine mass (Table 1).

Spontaneous tone of the PNA did not differ among groups (Table 1). However, PNA maximal diameters in young and old OVX+E2 were significantly greater than that of age-matched intact and OVX PNAs (Table 1).

### Endothelium-Dependent Vasodilator Responses

**Figure 1:**
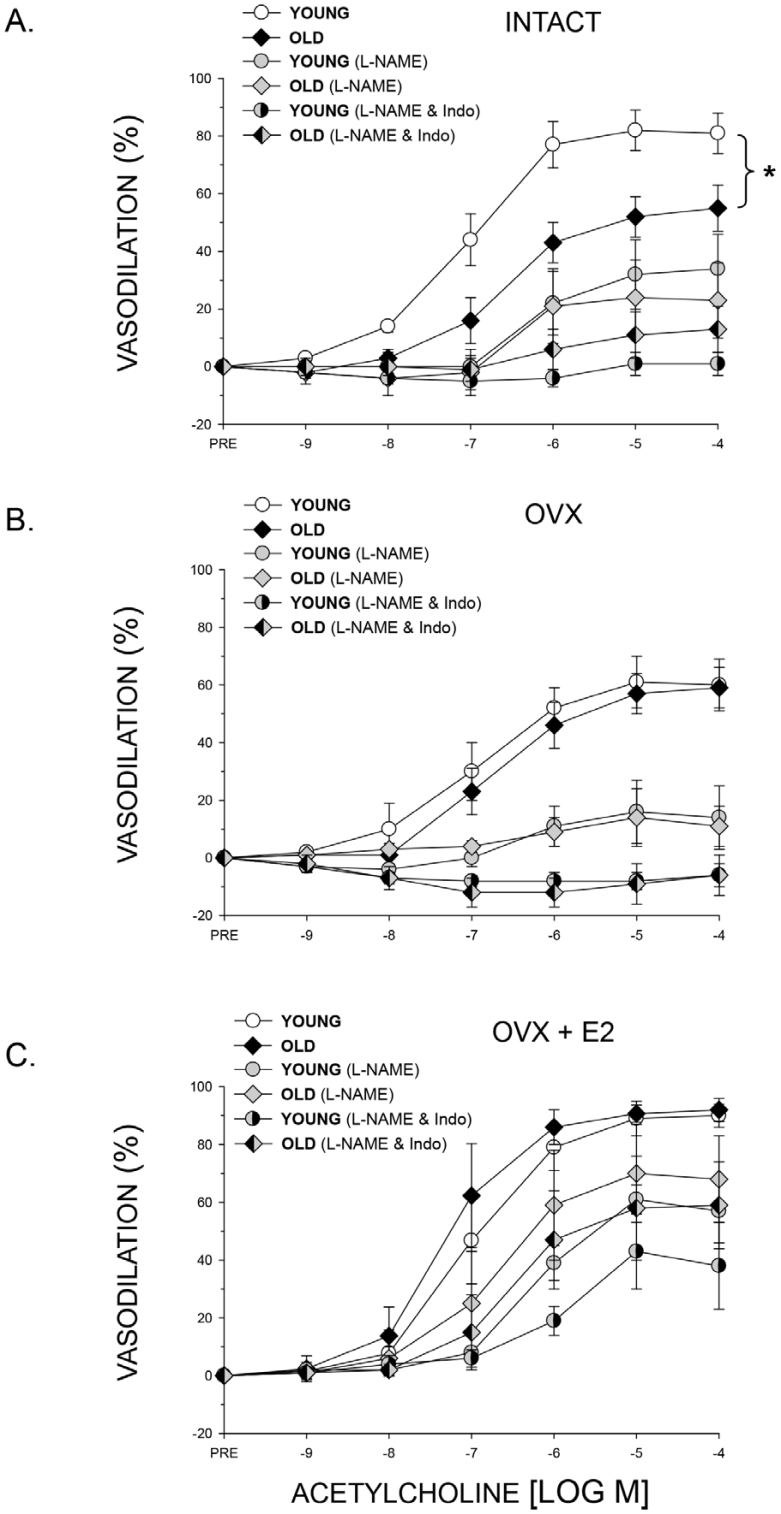
The effects of age, nitric oxide synthase inhibition with L-NAME and combined nitric oxide synthase and cyclooxygenase inhibition (L-NAME & Indo) on vasodilator responses of the PNA to increasing doses of the endothelium-dependent vasodilator acetylcholine. The PNA responses are determined in young and old A) intact, B) ovariectomized (OVX), and C) ovariectomized and estrogen replaced (OVX+E2) female rats. A. There was an age-related difference in vasodilator responses between young and old INTACT female rats. Blockade with L-NAME significantly reduced vasodilation in both groups (*P*<0.05) and abolished the age-related difference in the vasodilator response. The combined blockade with L-NAME and Indo further reduced PNA vasodilation in the young rats (*P*<0.05). B. There were no differences in the vasodilator response between young and old OVX rats under any of the three conditions. Blockade with L-NAME significantly reduced vasodilation in both groups (*P*<0.05), and the combined blockade further diminished vasodilation in both OVX groups (*P*<0.05). C. There were no differences in vasodilator responses between young and old OVX+E2 rats under any of the three conditions. Inhibition with L-NAME significantly reduced vasodilation in both groups (*P*<0.05), whereas the combined L-NAME & Indo blockade had no further effect than L-NAME alone. Values are means ± S.E; *n* = 9–11 animals per group. *Indicates the vasodilator response is significantly different between groups (*P*<0.05).

ACh-induced vasodilation of the femoral PNA was lower in old intact relative to that in young intact (Fig. 1A). Inhibition of the NOS pathway with L-NAME abolished differences in vasodilation between the two age groups (Fig. 1A). The combined inhibition of the NOS and COX pathways further lowered vasodilation of PNAs from young rats, and there remained no aging differences (Fig. 1A).

Ovariectomy eliminated the old age-related difference in PNA vasodilation (Fig. 1B) that was evidenced between young and old intact animals. L-NAME lowered vasodilator responses in PNAs from both young and old OVX rats, indicating that most of the vasodilator response occurred through the NOS signaling mechanism. The combination of L-NAME and Indo abolished all vasodilation in the PNA of OVX animals (Fig. 1B). Ovariectomy with estrogen replacement also showed no age-related difference in vasodilation between young and old rats (Fig. 1C). NOS inhibition lowered endothelium-dependent vasodilator responses in OVX+E2 rats, whereas the addition of the COX inhibitor Indo had little further effect in PNAs from both young and old rats (Fig. 1C).

**Figure 2:**
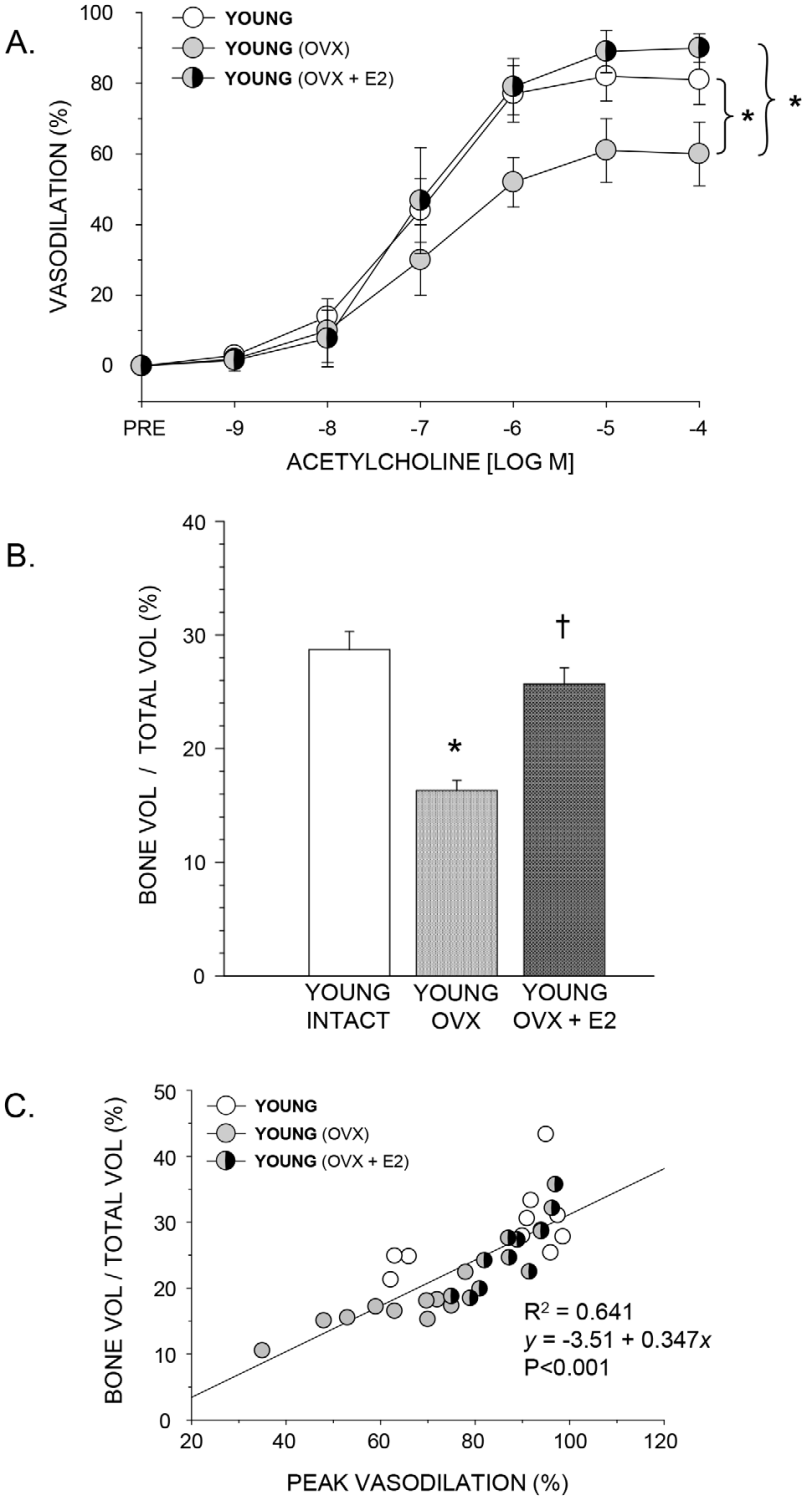
The effects of ovariectomy and estrogen replacement on PNA endothelium-dependent vasodilation and trabecular bone volume in the femur of young rats. A) acetylcholine-induced vasodilation of the PNA, B) trabecular bone volume (BV/TV) of the distal femur, and C) the relation between peak endothelium-dependent vasodilation (%) and trabecular bone volume (%) in young intact, ovariectomized (OVX), and ovariectomized and estrogen replaced (OVX+E2) rats. A. Vasodilation was significantly lower in OVX rats, while estrogen replacement (OVX+E2) maintained vasodilator function to levels similar to that in intact animals. *Indicates the vasodilator response is significantly different between indicated groups (*P*<0.05). B. Ovariectomy significantly reduced BV/TV vs. young intact rats, and OVX+E2 maintained trabecular bone volume (BV/TV) to levels equivalent to that of intact animals. *Indicates a significant difference from intact controls; ^†^indicates a significant difference from OVX rats. C. Regression analysis indicates a linear relation between peak endothelium-dependent vasodilation (%) and trabecular bone volume (%) of the distal femur (*P*<0.001). Values are means ± S.E; *n* = 9–11 animals per group.

**Figure 3:**
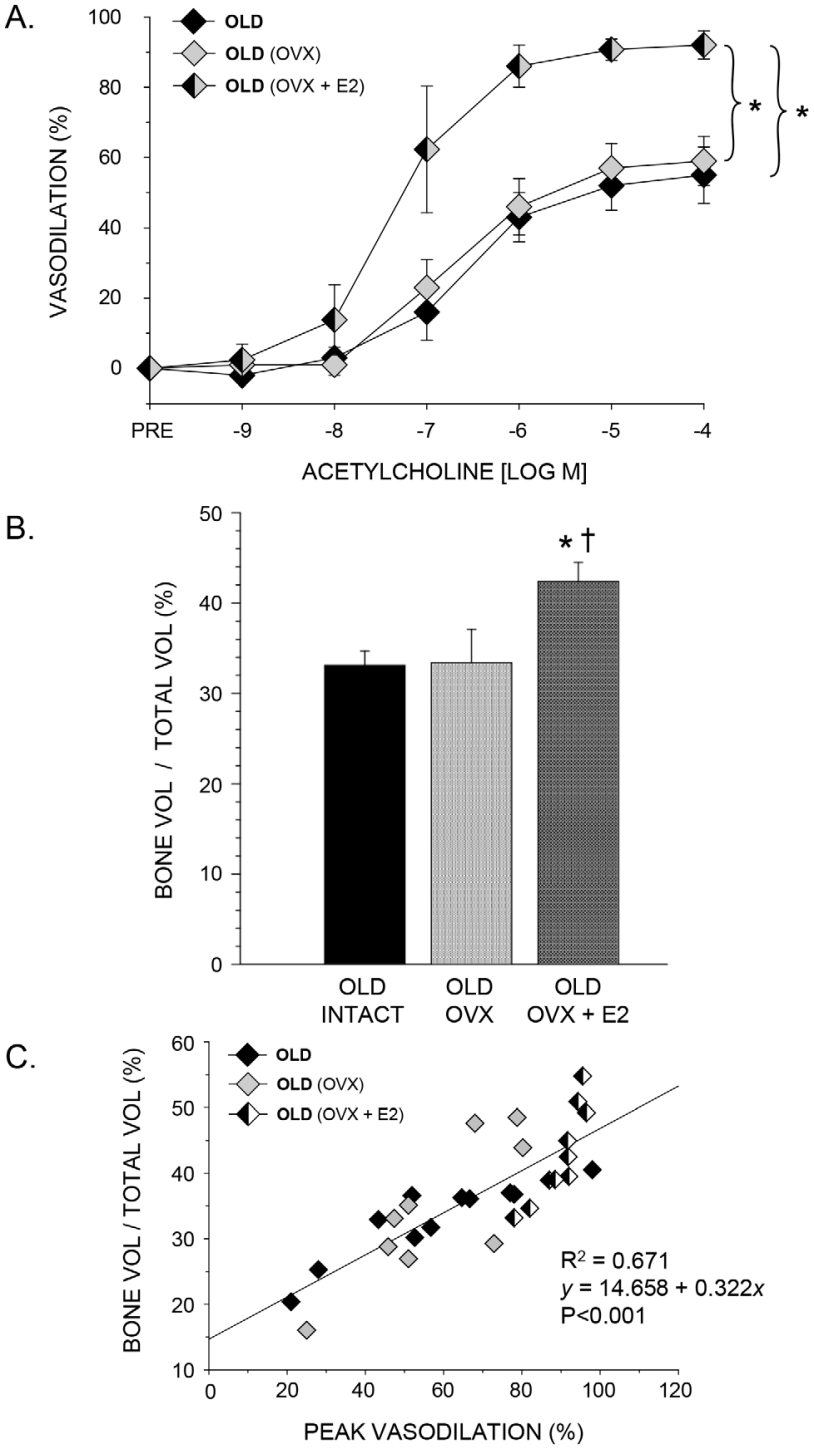
The effects of ovariectomy and estrogen replacement on PNA endothelium-dependent vasodilation and trabecular bone volume in the femur of old rats. A) acetylcholine-induced vasodilation of the PNA, B) trabecular bone volume (BV/TV) of the distal femur, and C) the relation between peak endothelium-dependent vasodilation (%) and trabecular bone volume (%) in old intact, ovariectomized (OVX), and ovariectomized and estrogen replaced (OVX+E2) rats. A. Vasodilation was not different between old intact control and old OVX rats, while estrogen replacement (OVX+E2) elevated endothelium-dependent vasodilation to levels above that of intact and OVX animals. *Indicates the vasodilator response is significantly different between indicated groups (*P*<0.05). B. Ovariectomy had no effect on BV/TV vs. old intact rats, whereas OVX+E2 elevated trabecular bone volume (BV/TV). *Indicates a significant difference from intact controls; ^†^indicates a significant difference from OVX rats. C. Regression analysis indicates a linear relation between peak endothelium-dependent vasodilation (%) and trabecular bone volume (%) of the distal femur (*P*<0.001). Values are means ± S.E; *n* = 9–11 animals per group.

In young rats, the lowering of estrogen levels with OVX lowered endothelium-dependent vasodilation, whereas estrogen repletion in OVX+E2 rats restored vasodilation to levels equivalent to that in young intact rats (Fig. 2A). In PNAs from old rats, OVX had no effect on endothelium-dependent vasodilator responses (Fig. 3A). The chronic addition of exogenous estrogen elevated vasodilation of PNAs from old rats (Fig. 3A) to levels that were similar to those in young animals (Fig. 1C).

### DEA NONOate Vasodilator Responses

**Figure 4:**
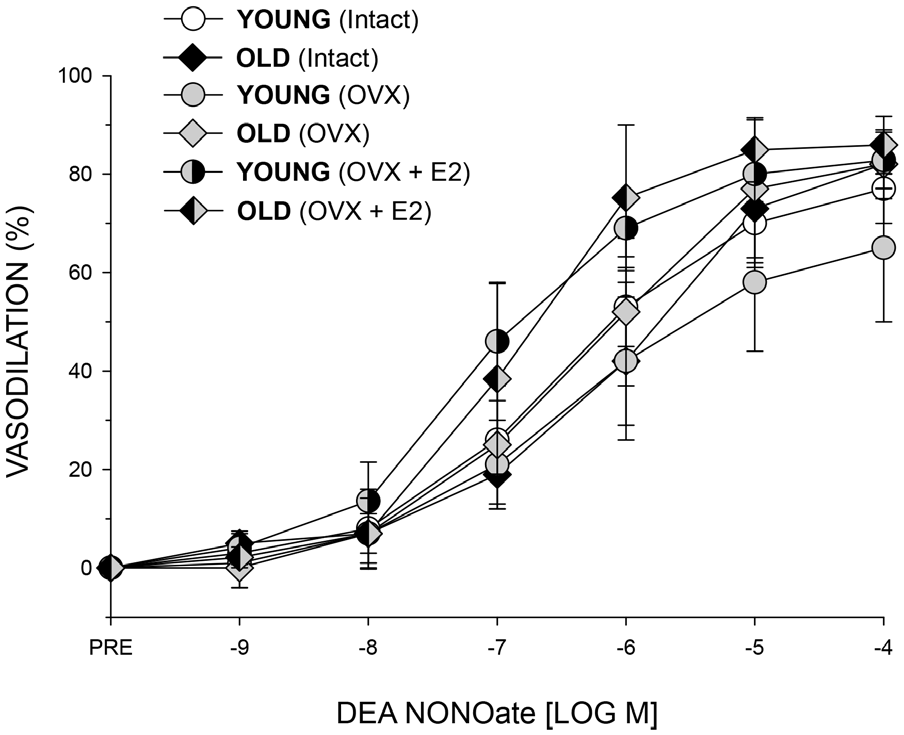
Effects of age, ovariectomy (OVX), and ovariectomy and estrogen replaced (OVX+E2) on vasodilator responses of the PNA to increasing doses of the endothelium-independent, direct NO donor DEA NONOate in female rats. Responses were not different among groups. Values are means ± S.E; *n* = 9–11 animals per group.

Vasodilation to the direct NO donor DEA NONOate was not different among groups (Fig. 4), indicating differences in ACh-induced vasodilation were mediated through the PNA endothelial cells and not differences in the responsiveness of the smooth muscle cells.

### Trabecular Bone Volume

In young rats, the lowering of plasma estrogen concentration through OVX reduced trabecular bone volume (BV/TV) by 43% compared to young intact (Fig. 2B). Estrogen replacement in OVX+E2 rats increased bone volume to levels similar to those in young intact rats (Fig. 2B). OVX in old rats had no effect on trabecular bone volume (Fig. 3B). However, the addition of exogenous estrogen resulted in 28% higher trabecular bone volume compared to controls (Fig. 3B).

### Regression Analyses

**Figure 5:**
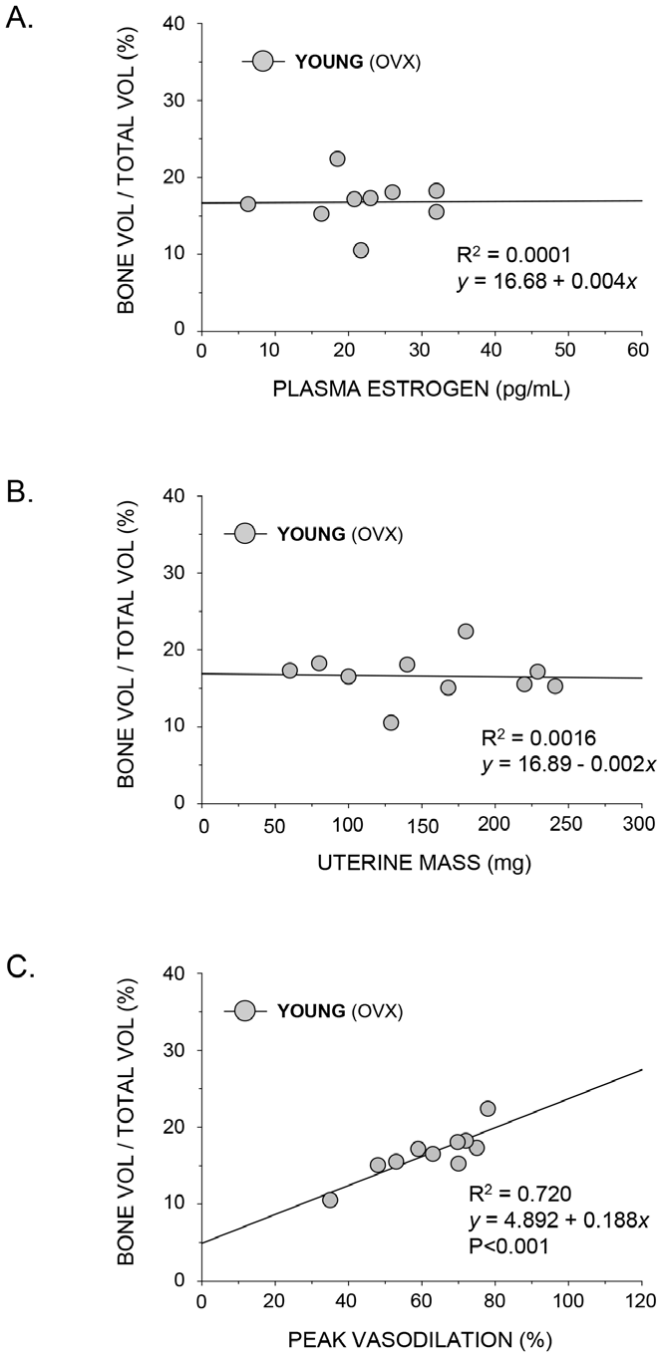
Scattergrams showing the relation between plasma estrogen, uterine mass and endothelium-dependent vasodilation on trabecular bone volume (BV/TV) in the distal femur of young ovariectomized rats. A) plasma estrogen concentration from animals with estrogen levels within the detectable range; B) uterine mass, a biomarker for reproductive hormone concentration ovariectomized; and C) peak endothelium-dependent vasodilation of the femoral PNA. BV/TV was not related to either plasma estrogen (A) or uterine mass (B) in this group, whereas a significant (P<0.001) linear relation exists between BV/TV and percent PNA vasodilation (C).

**Figure 6:**
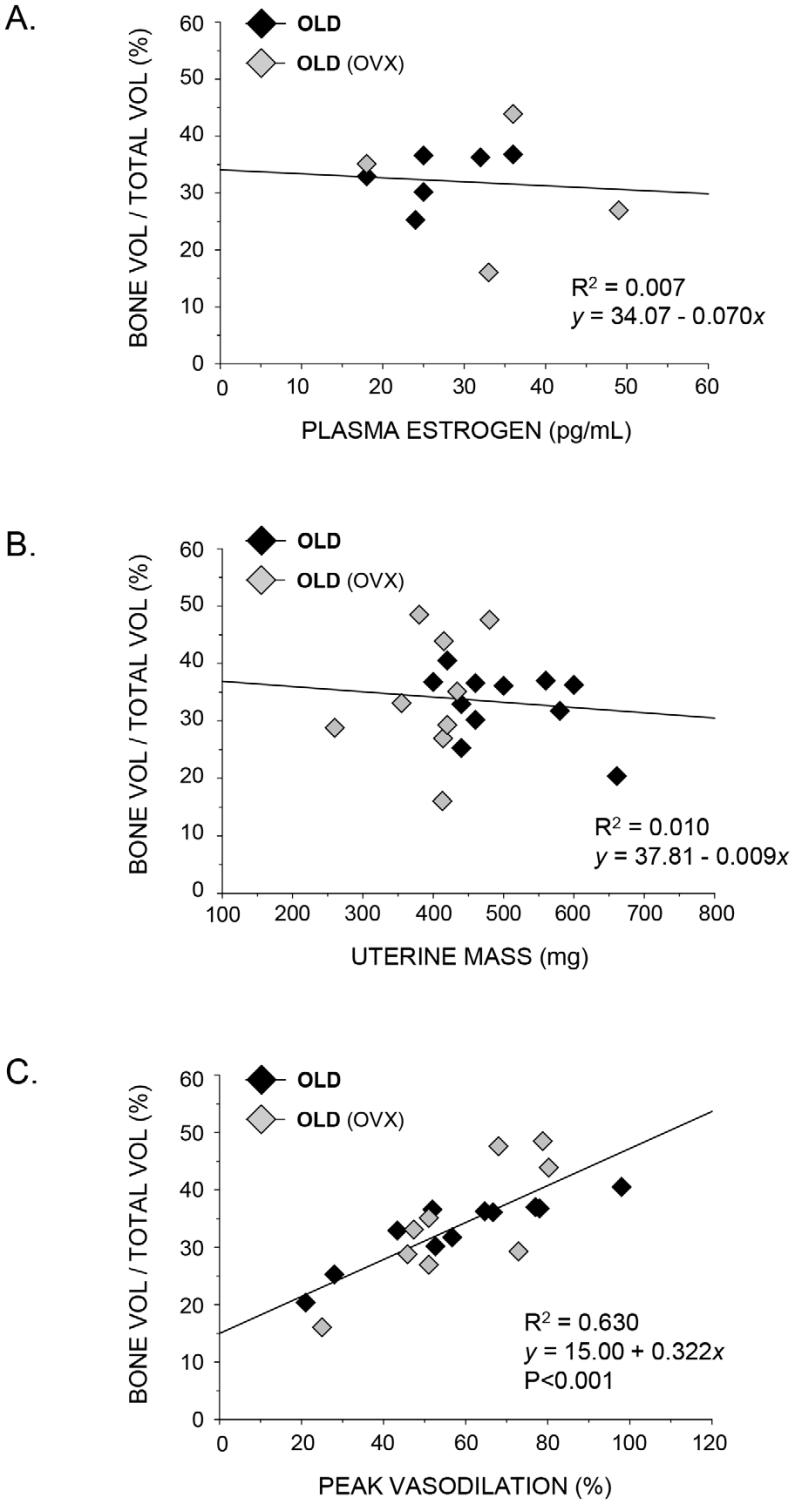
Scattergrams showing the relation between plasma estrogen, uterine mass and endothelium-dependent vasodilation on trabecular bone volume (BV/TV) in the distal femur of old intact and ovariectomized rats. A) plasma estrogen concentration from animals with estrogen levels within the detectable range; B) uterine mass, a biomarker for reproductive hormone concentration; and C) peak endothelium-dependent vasodilation of the femoral PNA. BV/TV was not related to either plasma estrogen (A) or uterine mass (B) in these groups, whereas a significant (P<0.001) linear relation exists between BV/TV and percent PNA vasodilation (C).

Trabecular bone volume was significantly correlated to peak vasodilation in both young and old rats (Fig. 2C and 3C) whereby regression analyses indicated R^2^ values of 0.641 and 0.671 in young and old rats, respectively. In addition, in rats with low estrogen status (i.e., young OVX, old intact and old OVX), there was no relation between trabecular bone volume and plasma estrogen (Figs. 5A and 6A) or uterine mass (Figs. 5B and 6B). However, these groups displayed a significant relation between peak endothelium-dependent vasodilation and trabecular bone volume (Figs. 5C and 6C).

## Discussion

The purpose of this study was to determine whether estrogen status affects endothelium-dependent vasodilation in the femoral diaphyseal principal nutrient artery, and whether these putative changes in vascular endothelial function are associated with alterations in trabecular bone volume. In young rats, the results demonstrate that lowering circulating estrogen through ovariectomy impairs endothelium-dependent vasodilation (Fig. 2A) and diminishes trabecular bone volume in the femur (Fig. 2B), whereas replacement of estrogen following ovariectomy maintains both endothelium-mediated vasodilation and trabecular bone volume. In old rats, ovariectomy had no effect on circulating estrogen levels, endothelium-dependent vasodilation (Fig. 3A) or trabecular bone volume (Fig. 3B). However, the addition of exogenous estrogen following ovariectomy elevated endothelium-dependent vasodilation (Fig. 3A) and increased bone volume (Fig. 3B). The estrogen-induced alterations in endothelium-mediated vasodilation were strongly correlated with changes in trabecular bone volume in both young (Fig. 2C) and old (Fig. 3C) rats. To our knowledge, these are the first data to report the effects of ovariectomy and estrogen replacement on endothelium-dependent vasodilation in bone from young and old animals, and the first to examine the relation between vascular function and bone properties with age and estrogen status as variables.

It has long been appreciated that estrogen can have positive effects on the skeletal system. For example, during adolescent growth estrogen is the critical reproductive factor for both girls and boys [31]. Additionally, a fall in estrogen concentration below 30 pg/mL during menopause corresponds to the period of accelerated bone loss [31]. The loss of estrogen following menopause creates an imbalance between bone resorption and formation that leads to a net loss of bone [32]. Many postmenopausal women exhibit reduced bone mass as well as high-turnover bone metabolism, and the reversal of these conditions are observed following estrogen replacement [33]. The preservation of bone mass during this period requires exogenous administration of estrogen sufficient to raise plasma concentrations to 50 pg/mL [31]. Estrogen receptors are present on osteoblasts [34], [35], but the actions of estrogen on these cells and osteoblast-like cells have been variable [4]. A recent study suggests that the mechanism(s) of action of estrogen on bone cell activity is related to the control of function and lifespan of osteoclasts, which serves to ultimately minimize bone resorption [33]. The exact mechanism(s) through which estrogen acts to modulate bone cell activity is not completely understood, allowing for the possibility that non-skeletal tissue may act to modulate bone cell activity [31]. We hypothesize that one such mechanism is related to bone vascular endothelial function and its ability to 1) directly influence bone cell activity through local release of endothelium-derived signaling molecules such as NO and PGI_2_ [36], [37], and 2) to regulate skeletal perfusion, which could indirectly affect bone cell activity through its impact on fluid filtration out of the vascular compartment and, correspondingly, interstitial fluid pressure in bone [13], [37]–[40].

There is a growing body of literature that supports the notion that the bone vasculature can modulate bone cell activity [40]–[42]. In regard to old age-related bone loss, previous studies in humans and animals have shown compromised bone and marrow blood flow are associated with osteopenia [18]–[22] and studies in male rats have shown impaired bone vascular endothelial function is related to old age-induced bone loss [21], [22]. Additionally, a recent study has reported that a program of low-intensity endurance exercise training that elicits low mechanical strain on bone restores endothelial NO-mediated vasodilation of the PNA and increases trabecular bone volume and mineral density in the distal femur of old male rats [21]. The results demonstrated that old age-induced decrements and training-induced enhancement of NO-mediated vasodilation of the bone vasculature are positively correlated with changes in the bone properties [21], [22].

Results from the present study also demonstrate an association between NO-mediated vasodilation of bone arteries and trabecular bone volume when estrogen status is altered through ovariectomy and estrogen replacement (Fig. 2C and 3C). Although these findings are consistent with the general hypothesis that bone vascular endothelial function is coupled to bone remodeling activity, these results alone are inconclusive since estrogen may have direct effects on both the bone vasculature and bone cell activity, which could be independent of each other. Therefore, we sought to determine whether a relation exists between vascular endothelial function and bone volume in animals where estrogen concentration was low, i.e., the young OVX and the old intact and OVX groups. Regression analyses indicated that there were no relations (P>0.05) between trabecular bone volume and plasma estrogen (Figs. 5A and 6A) or uterine mass (Figs. 5B and 6B), a biomarker for reproductive hormone status, indicating that the direct effects of estrogen on cancellous bone volume are negligible in these low-estrogen groups. However, there was a significant association between peak endothelium-dependent vasodilation and trabecular bone volume (Figs. 5C and 6C) in the femur. These findings provide support for the concept of a vascular coupling mechanism in bone. This conclusion of a coupling mechanism between the bone vasculature and bone mass is further supported by two studies in humans, where forearm conduit artery endothelial vasodilator responsiveness, used as a surrogate measure of bone endothelial function, was positively correlated with lumbar spine bone mineral density in postmenopausal women [43], [44]. Thus, in groups without the potentially confounding influence of estrogen, the relation between endothelial function and bone mass is still evident.

Intact young and old female rats regulate vasodilation of the femoral PNA primarily through a NOS signaling mechanism and to a lesser extent through a COX signaling pathway (Fig. 1A). These regulatory mechanisms are similar to those of male animals, whereby endothelium-dependent vasodilation is mediated predominantly via the NOS signaling cascade [21], [22]. The old age-associated decrement in endothelium-dependent vasodilation in female rats results from declines in NO-mediated regulation, which is also similar to that observed in male rats. In the absence of estrogen (OVX group), vasodilation in the presence of L-NAME alone is eliminated (Fig. 1B). Interestingly, with estrogen replacement the regulating vasodilator mechanism shifts from the primarily NO signaling pathway to one that includes NO and the putative endothelium-derived hyperpolarizing factor, as indicated by the significant vasodilation that occurs in estrogen replete rats when both NOS and COX signaling pathways are blocked (Fig. 1C). Therefore, the addition of estrogen from an exogenous source does not simply restore the regulatory mechanisms for vasodilation but alters the signaling mechanisms through which the peak endothelium-dependent vasodilation of the bone resistance vasculature is achieved. A shift in the mechanism of endothelium-dependent vasodilation with OVX has also been reported in mesenteric arteries. In intact rats, endothelium-dependent vasodilation of mesenteric arteries is mediated through both the NOS and COX signaling pathways; OVX diminished the NO-mediated component and augmented the contribution of the COX signaling pathway [45].

The decrement in endothelium-dependent vasodilation of the femoral PNA with OVX in young rats has also been reported to occur in other vascular beds. For example, OVX has been shown to depress endothelium-dependent vasodilation of pulmonary arteries [46], the thoracic aorta [46], and coronary resistance arteries [26] in young rats. However, several studies examining the effects of OVX on endothelium-dependent vasodilation in bone arteries of young animals have reported results that are dissimilar to those of the present investigation. In the first study, arterial segments from rabbits were isolated from the metaphyseal cancellous bone and the diaphyseal bone marrow of the femur six weeks after OVX [47]. The vessel segments with a diameter of ∼250 µm were cut into rings and two 40 µm diameter steel wires were passed through the lumen of the vessel rings. Changes in isometric force were measured with increasing concentrations of ACh after the arterial rings were preconstricted with norepinephrine. The results indicated that OVX did not alter endothelium-dependent relaxation [47]. The disparity of results compared to the current study could be due to several factors, including differences in 1) the animal species studied and 2) the techniques employed to study the vascular responses. For instance, unlike rats and humans, rabbits do not undergo the various stages of the estrous cycle [48]. Also, the wire myograph technique used is not ideal for determination of endothelium-dependent vasodilation in small diameter vessels because of the potential for the wires to damage endothelial cells as they are passed through the lumen of the vessel rings.

The second study with disparate results relative to those of the current study investigated the effects of OVX in medullary arterioles from the femur of rats using the pressurized vessel technique [49]. Vasodilation induced by ACh was not different between medullary arterioles from intact and OVX rats. The discrepancy in findings may result from differences in the strain of rat studied (Fischer-344 vs. Sprague Dawley), age of the animals at sacrifice (6 and 24 months vs. 3 to 3 ½ months) and, most probably, differences in the period of OVX (2 months vs. 7–10 days).

Few studies have examined the effects of OVX on endothelium-dependent vasodilation with advanced age. Castillo et al. [50] reported that relaxation of aortic rings to ACh was reduced in 20-month-old OVX rats. In contrast, OVX had no further effect to depress endothelium-dependent vasodilation of coronary resistance arteries [26] and femoral PNAs (present study) from old rats. These results indicate that the old age-associated decline in circulating estrogen may contribute to the overall reduction in endothelial cell vasodilator responsiveness that occurs with advancing age [21], [22], [27], [29].

The lack of bone loss with ovariectomy in the aged animals was counterintuitive to anticipated results and other published reports. For example, ovariectomy in rats 18 months of age has shown volume decrements in long bones as well as the vertebrae [51]–[53]. However, to our knowledge, there are no published reports on bone responses to ovariectomy utilizing rats of such an advanced age (i.e., 24 months) as the current investigation.

In conclusion, the results of the present study demonstrate that lowering circulating estrogen through ovariectomy impairs endothelium-dependent vasodilation (Fig. 2A) and diminishes trabecular bone volume (Fig. 2B) in the femur of young, but not in old female rats (Figs. 3A and 3B). Estrogen replacement following ovariectomy maintains and enhances both endothelium-mediated vasodilation and trabecular bone volume in young and old animals, respectively. The estrogen-induced alterations in endothelium-mediated vasodilation were strongly correlated with changes in trabecular bone volume in both young (Fig. 2C) and old (Fig. 3C) rats, indicating a possible coupling mechanism between the vascular endothelium and bone remodeling activity. Unfortunately, these results alone are inconclusive due to the fact that estrogen may have direct effects on both the bone vasculature and bone cell activity that are independent of each other. However, in young OVX and old intact and OVX animals, where circulating estrogen levels are low with negligible effects on cancellous bone volume (Figs. 5A, 5B, 6A and 6B), regression analysis demonstrated a significant relation between peak endothelium-dependent vasodilation and trabecular bone volume (Figs. 5C and 6C) in the femur. These results provide support for a mechanism coupling vascular endothelial vasodilator signaling with bone volume.
